# Contamination by Norovirus and Adenovirus on Environmental Surfaces and in Hands of Conscripts in Two Finnish Garrisons

**DOI:** 10.1007/s12560-016-9262-4

**Published:** 2016-09-30

**Authors:** Satu Oristo, Maria Rönnqvist, Mika Aho, Ava Sovijärvi, Tuula Hannila-Handelberg, Ari Hörman, Simo Nikkari, Paula M. Kinnunen, Leena Maunula

**Affiliations:** 1 Department of Food Hygiene and Environmental Health, Faculty of Veterinary Medicine, University of Helsinki, Agnes Sjöbergin katu 2, 00790 Helsinki, Finland; 2 Finnish Food Safety Authority Evira, Mustialankatu 3, 00790 Helsinki, Finland; 3 Centre for Military Medicine, Tukholmankatu 8 A, 00290 Helsinki, Finland; 4 Finnish Defence Command Logistics Division, Fabianinkatu 2, 00130 Helsinki, Finland

**Keywords:** Adenovirus, Environmental contamination, Gastroenteritis, Norovirus, Questionnaire, Surface swab

## Abstract

This study investigated the presence of norovirus and adenovirus, especially enteric adenovirus, on the environmental surfaces (*n* = 481) and military conscripts’ hands (*n* = 109) in two Finnish garrisons (A and B) in 2013 and 2014. A questionnaire study was conducted to reveal possible correlations between viral findings on the conscripts’ hands and their acute gastroenteritis symptoms. In addition to the swab samples, 14 fecal samples were obtained for viral analysis. In total, norovirus was present in 9.0 % of the surface swabs in 2013, whereas enteric adenovirus was present in 0.0 % and non-enteric adenovirus in 9.4 %. In the same year, 2.6 % of the hand swabs contained norovirus, 2.6 % enteric adenovirus, and 40.3 % non-enteric adenovirus. Norovirus GI.6 was continually detected on the surfaces of garrison A, and identical virus was detected in some of the fecal samples. In garrison B, two slightly different norovirus GII.4 strains were present on the surfaces. The questionnaires revealed no recent acute gastroenteritis cases in garrison A, but in garrison B, where the norovirus-positive hand swabs were collected, 30.6 % of the conscripts reported of recent symptoms. In 2014, norovirus was rarely detected, but adenovirus was again frequently present, both on the surfaces and hands. Taken together, our results suggest that gastroenteritis outbreaks occurred in 2013, but not in 2014. Due to the low number of hand swabs positive for enteric viruses, no conclusions about associations between viral findings and gastroenteritis symptoms could be drawn. This study increased our understanding of the possible transmission of viruses via contaminated environment and hands.

**Electronic supplementary material:**

The online version of this article (doi:10.1007/s12560-016-9262-4) contains supplementary material, which is available to authorized users.

## Introduction

Norovirus (NoV) is the leading cause of acute viral gastroenteritis in all age groups, as it has been reported to be responsible for almost 20 % of all acute gastroenteritis (AGE) cases worldwide (Ahmed et al. 2014). Several NoV genotypes are recognized among the three genogroups (GI, GII, GIV) that infect humans. Each genotype possesses a characteristic set of epidemiological and clinical features (Matthews et al. 2012; Kirby et al. 2014). Clinical manifestations of NoV infection are typically vomiting, abdominal cramps, and diarrhea, but viral shedding can also be asymptomatic (Teunis et al. 2015). The infectious dose of NoV is low (Atmar et al. 2008; Teunis et al. 2008) and the virus exploits several transmission routes. It spreads efficiently, especially in semi-closed settings; during a NoV outbreak in a scout camp setting, it was estimated that 14 secondary cases occurred per every primary case, when enhanced hygienic measures were not practiced (Heijne et al. 2009).

Over 60 adenovirus (AdV) types are recognized to date (Robinson et al. 2013), and in addition to respiratory disease, different AdV types are capable of causing meningitis, eye infections, and gastroenteritis (Lynch et al. 2011). Respiratory AdV infections have affected the armed forces so severely in the past that an efficient vaccine against the most common types of AdV responsible for respiratory disease (types 4 and 7) is routinely used in the US Armed Forces (Radin et al. 2014). AdV types 40 and 41 are known as enteric AdVs (eAdVs), as they are the most common types associated with gastroenteritis (Lynch et al. 2011). Although clinical gastroenteritis due to eAdV usually only occurs in children and immunocompromised people (Lynch et al. 2011), they are so common in the general population that they have been proposed as viral markers of fecal contamination of water (Rusiñol et al. 2014).

Both NoV and AdV infections are problematic for the armed forces because these are capable of causing a remarkable reduction in the operational efficiency of the affected units. The aim of this study therefore was to characterize the contamination by NoV and AdV, especially eAdV, on environmental surfaces and army conscripts’ hands in military garrison settings. Hand swabbing was coupled with a questionnaire to reveal any correlation between viral findings on conscripts’ hands and their AGE symptoms, or other signs of a possible AGE outbreak. In 2013, the sampling was performed in March–May, when NoV outbreaks typically occur (Kroneman et al. 2008). In 2014, the sampling was done earlier, in January–February, in order to follow the possible transmission of NoV among the new conscripts during their first training period.

## Materials and Methods

### Surface Swab Sampling

**Fig. 1:**
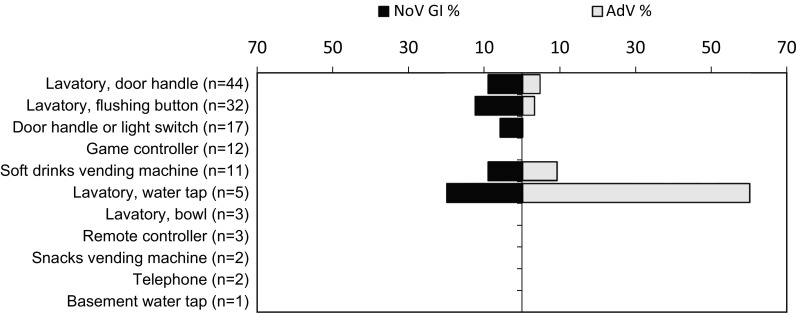
Distribution of norovirus (NoV) and adenovirus (AdV) findings over different surface swabbing sites in garrison A in 2013. All NoV findings represented genogroup I (NoV GI)

**Fig. 2:**
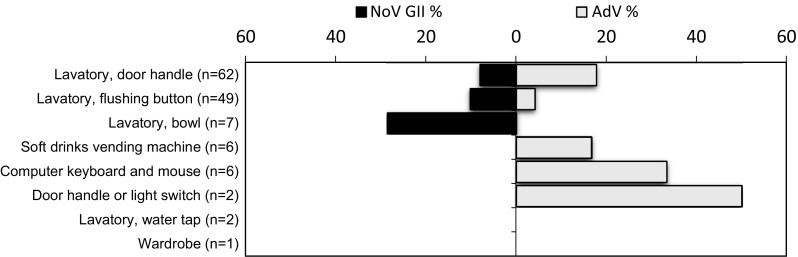
Distribution of norovirus (NoV) and adenovirus (AdV) findings over different surface swabbing sites in garrison B in 2013. All NoV findings represented genogroup II (NoV GII)

In March–May 2013, we collected 132 surface swabs in garrison A, and 135 surface swabs in garrison B, during six visits to each garrison (Table 1). In addition, 214 surface swabs were collected during 11 visits to garrison B in January–February 2014. The swabbing was performed as previously described by Rönnqvist et al. (2013). Briefly, a 25 cm^2^ surface area (or the whole object in case it was smaller) was swabbed with a polyester or microfiber swab moistened in phosphate-buffered saline (PBS). The swabs were taken from surfaces that are often touched, e.g., door handles, flushing buttons, vending machines, and electronic devices. Most of the sampling sites within both garrisons were in the lavatories (76.4 % in 2013 and 74.3 % in 2014), but swab samples from frequently touched objects in the conscripts’ living quarters were also included (Table 1; Figs. 1, 2).

**Table 1:** A summary of the different samples collected in garrisons A and B during the study period

Sample category	Year		
	2013		2014
	Garrison A	Garrison B	Garrison B
Surface swabs			
No. of swabs per sampled building			
Health center	22	22	6
Barracks	110	107	208
Cafe	0	6	0
Total	132	135	214
Sampling period (no. of visits)	Mar.12–May 14 (6)	Apr.10–May 22 (6)	Jan.3–Feb.2 (11)
Hand swabs			
No. of hand swabs	28	49	32
Sampling period (no. of visits)	Apr.16–Apr.23 (2)	Apr.17–May 8 (3)	Jan.29–Feb.2 (2)
Fecal samples			
No. of fecal samples	11	0	3
Sampling period^a^	Mar.5–May 14	–	Jan.30–Feb.4

^a^The 11 fecal samples collected in 2013 were available for viral analysis only after the surface and hand swabbing period was finished in May 2013

### Hand Swab Sampling

We collected 28 hand swabs during two of the visits to garrison A in April 2013, and 49 hand swabs during three of the visits to garrison B in April–May 2013 (Table 1). The conscripts who participated in the hand swab study were randomly selected in the sick bay waiting area. Of the garrison A and B conscripts, 8/28 (28.6 %) and 25/49 (51.0 %), respectively, resided in the barracks from where the surface swabs were taken. In January–February 2014, 32 hand swabs were collected during two of the visits to garrison B. In contrast to the hand swab study performed in 2013, the garrison B conscripts who participated in 2014 were all residing in the sampled barracks. Hand swabbing was performed similarly to the surface swabbing but using only the microfiber swab. Both palms were swabbed for at least 1 min.

### Questionnaires

All the conscripts (*n* = 109) who participated in the hand swab study filled in a questionnaire, in which they reported when they had last experienced AGE symptoms (diarrhea and either abdominal pain, vomiting, or both) and whether they had been in contact with other conscripts or non-military persons who had AGE symptoms within the previous 6 days. Although the participants for the hand swab study were selected in the sick bay waiting area in 2013, their reason for visiting there on the sampling date was not enquired. The hand swabs and questionnaires were collected anonymously.

### Fecal Samples

Our sampling scheme included the collection and analysis of only swab samples, but after the surface and hand swab sampling period was finished in 2013, we obtained 11 anonymous fecal samples from conscripts who had suffered from gastroenteritis in garrison A between March 5, and May 8, 2013 (Table 1). These samples were collected by the health care personnel of garrison A. In garrison B, no fecal samples were collected in 2013 but three were obtained in 2014.

### Swab and Fecal Sample Preparation

A known amount of either murine norovirus (MuNoV) strain MNV-1 (kindly gifted by Professor Herbert W. Virgin, Washington University, St. Louis, MO, USA) or mengovirus (MeV) strain M_C0_ (kindly gifted by Professor Albert Bosch, University of Barcelona, Spain) was added directly on the surface and hand swabs to act as a process control. Approximately every 12th swab sample was spiked with 1.0 × 10^5^ PCR units (PCR-u) of MuNoV in 2013, so that at least one spiked sample was included in each nucleic acid extraction batch. In 2014, every 6th swab sample was spiked either with 2.0 × 10^4^ or 2.0 × 10^5^ PCR-u of MeV. The viral particles were eluted from the swabs by a semi-direct lysis method, and the nucleic acids were extracted as previously described (Rönnqvist et al. 2013). 10 % fecal suspensions were prepared in sterile 1 x PBS, and nucleic acids were extracted with the QiaAmp Mini Viral RNA kit (QIAGEN, Hilden, Germany) according to the manufacturer’s instructions.

### Real-Time Reverse Transcription PCR and PCR Protocols

The swab and fecal samples were screened for NoV GI and GII by real-time reverse transcription PCR (rRT-PCR), whereas real-time PCR (rPCR) was used for screening AdV. All primers and probes used in this study are presented in Online Resource 1. NoV GII detection was performed as previously described (Rönnqvist et al. 2013), and the same protocol was used for NoV GI, except 0.9 µM of each GI-specific primer and 0.3 µM of GI-specific probe were used. MuNoV and MeV were analyzed by a similar method to NoV GII but with virus-specific primers and probes. The QuantiTect Probe PCR kit (QIAGEN) was used both for the detection of all AdVs and then for the detection of eAdV in the AdV-positive samples. The 20 µl AdV (or eAdV) reaction mix consisted of 10 µl of 2 x QuantiTect Probe PCR Master Mix, 1.0 µM of reverse and forward primers, 0.2 µM of probe, 0.6 µl of PCR-grade H_2_O, and 5 µl of template. Initial activation was performed at 95 °C for 15 min, followed by 45 cycles of 94 °C for 15 s, 55 °C for 45 s, and 72 °C for 45 s. Both rRT-PCR and rPCR reactions were performed using the Rotor-Gene 3000 thermal cycler (QIAGEN). All viral findings were immediately reported to the respective garrisons’ personnel.

### Reverse Transcription PCR Protocols

The samples that were positive for NoVs by rRT-PCR were subjected to reverse transcription PCR (RT-PCR) reactions, performed with the QIAGEN One-Step RT-PCR kit reagents (QIAGEN). Four different primer pairs that targeted the polymerase (ORF1) and/or the capsid (ORF2) region were used (Online Resource 1). Amplified products were visualized on 1.5 % SeaKem LE (Lonza, Basel, Switzerland) agarose gel with ethidium bromide staining, and sequenced according to the Sanger sequencing method in the Institute of Biotechnology, University of Helsinki, Finland.

### Data Analyses

Raw sequence data were analyzed using BioEdit software version 7.0.5.3 (http://www.mbio.ncsu.edu/BioEdit/bioedit.html) and sequence identities calculated using the Clustal Omega software version 1.2.1 (http://www.ebi.ac.uk/Tools/msa/clustalo/). The sequences were genotyped using the RIVM norovirus genotyping tool (Kroneman et al. 2011) (http://www.rivm.nl/mpf/norovirus/typingtool) and NCBI BLAST (http://blast.ncbi.nlm.nih.gov/Blast.cgi). IBM SPSS Statistics software version 22 (IBM Corp., Armonk, NY, USA) and OpenEpi version 3.03a (http://openepi.com/Menu/OE_Menu.htm) were used for statistical analyses of the results. *P* values < 0.05 were considered to be statistically significant.

## Results

### NoV and AdV Detection on the Environmental Surfaces of Garrisons A and B in 2013

In total, NoV was present in 9.0 % of the surface swabs collected in garrisons A and B in 2013, whereas eAdV was present in 0.0 % and non-eAdV in 9.4 %.

NoV GI was detected in garrison A in 9.1 % (12/132) of the surface swabs (Table 2). Most of the NoV-positive samples were collected from the lavatories (Fig. 1), but the difference between NoV findings for every garrison A lavatory (10.7 %; 9/84) and other surface (6.3 %; 3/48) was not significant. One of the sampled lavatory surfaces tested positive for NoV GI in two consecutive visits 4 weeks apart. None of the AdV findings on the surfaces of garrison A were confirmed as eAdV. Non-eAdV findings on the garrison A surfaces (6.1 %; 8/132) were similarly distributed between the lavatories and the other environmental surfaces (7.1 %; 6/84 vs. 4.2 %; 2/48) as for NoV, but none of the swabs were positive for both viruses. Non-eAdV was once detected twice on the same lavatory surface in two consecutive visits 1 week apart.

**Table 2:** Viral findings on the surface and hand swabs collected in garrison A during the study period

Sampling date	Garrison A			
	Surface swabs (%)		Hand swabs (%)	
	Norovirus^a^	Adenovirus^b^	Norovirus	Adenovirus
Year 2013				
Mar.12	8/30 (26.7)	3/30 (10.0)	–	–
Apr.9	2/20 (10.0)	0/20 (0.0)	–	–
Apr.16	1/21 (4.8)	4/21 (19.0)	0/16 (0.0)	7/16 (43.8)
Apr.23	0/18 (0.0)	1/18 (5.6)	0/12 (0.0)	7/12 (58.3)^c^
May 7	1/21 (4.8)	0/21 (0.0)	–	–
May 14	0/22 (0.0)	0/22 (0.0)	–	–
Total	12/132 (9.1)	8/132 (6.1)	0/28 (0.0)	14/28 (50.0)

^a^All detected noroviruses on the surfaces of this garrison belonged to genogroup I; of these norovirus-positive samples, three were confirmed as genotype GI.6 by sequencing

^b^None of the adenoviruses detected on the surfaces were confirmed as adenovirus type 40/41

^c^Two adenovirus strains detected on the hand swabs represented adenovirus type 40/41

NoVs were detected in garrison B on three sampling visits, but in contrast to garrison A, all strains belonged to the GII genogroup (8.9 %; 12/135) (Table 3), and all the NoV-positive swabs were collected from the lavatories (Fig. 2). One of the sampled lavatory surfaces tested positive for NoV GII in two consecutive visits 1 week apart. As in garrison A, none of the swabs were positive for eAdV or both NoV and AdV. Non-eAdV was again a frequent finding (12.6 %; 17/135), both on the lavatory (10.8 %; 13/120) and the other surfaces (26.7 %; 4/15). Three of the surfaces were non-eAdV-positive in two consecutive visits (twice the computer keyboard and once a door knob in the sick bay).

**Table 3:** Viral findings on the surface and hand swabs in garrison B during the study period

Sampling date	Garrison B			
	Surface swabs (%)		Hand swabs (%)	
	Norovirus^a^	Adenovirus^b^	Norovirus^a^	Adenovirus^b^
Year 2013				
Apr. 10	6/24 (25.0)	3/24 (12.5)	–	–
Apr.17	5/25 (20.0)	2/25 (8.0)	1/27 (3.7)	8/27 (29.6)
Apr.24	0/21 (0.0)	7/21 (33.3)	0/13 (0.0)	7/13 (53.8)
May 8	0/21 (0.0)	1/21 (4.8)	1/9 (11.1)	4/9 (44.4)
May 15	1/22 (4.5)	0/22 (0.0)	–	–
May 22	0/22 (0.0)	4/22 (18.2)	–	–
Total	12/135 (8.9)	17/135 (12.6)	2/49 (4.1)	19/49 (38.8)
Year 2014				
Jan.3	0/21 (0.0)	2/21 (9.5)	–	–
Jan.9	1/21 (4.8)	2/21 (9.5)	–	–
Jan.13 and Jan.16	0/44 (0.0)	1/44 (2.3)	–	–
Jan.21 and Jan.23	0/44 (0.0)	2/44 (4.5)	–	–
Jan.27, Jan.29, and Feb.2	0/64 (0.0)	2/64 (3.1)	0/32 (0.0)	6/32 (18.8)
Feb.5 and Feb.7	0/20 (0.0)	2/20 (10.0)	–	–
Total	1/214 (0.5)	11/214 (5.1)	0/32 (0.0)	6/32 (18.8)

^a^All detected noroviruses on the surfaces and hands in this garrison belonged to genogroup II; of these norovirus-positive samples, five were confirmed as genotype GII.4 by sequencing

^b^None of the detected adenoviruses were confirmed as adenovirus type 40/41

### NoV and AdV Findings in the Hand Swab Samples in Garrisons A and B in 2013

We collected a total of 77 hand swabs during two of the visits to garrison A and three of the visits to garrison B. Of these, 2.6 % (2/77) contained NoV, 2.6 % (2/77) eAdV and 40.3 % (31/77) non-eAdV.

The hand swabs of garrison A were all negative for NoV (Table 2). eAdV was, however, detected in 7.1 % (2/28) and non-eAdV in 42.9 % (12/28) of the hand swab samples. Two of the hand swabs collected in garrison B (4.1 %; 2/49) were positive for NoV GII (Table 3). Non-eAdVs were present in 38.8 % (19/49) of the hand swabs. NoV GII and non-eAdV were detected in the same hand swab sample on one occasion.

### NoV and AdV Detection in the Fecal Samples Collected in Garrison A in 2013

After we had finished analyzing the swab samples in 2013, we obtained 11 fecal samples for NoV and AdV analysis (Table 1). Three (33.3 %) of the nine fecal samples collected in the sick bay in the beginning of March 2013 were found to be positive for NoV GI, and two (22.2 %) were positive for NoV GII. All these NoV-positive fecal samples were collected less than a week before the first surface swabbing visit in March 12, 2013, when the number of NoV-positive surface samples (26.7 %; 8/30) was highest. The two fecal samples that were collected later, on March 13 and May 14, were NoV negative. All 11 samples were AdV-negative.

### NoV and AdV Findings in January–February 2014 (Garrison B Only)

One lavatory surface tested positive for NoV GII (0.5 %; 1/214) in 2014 (Table 3). AdVs, all non-eAdVs, were detected in 4.4 % (7/159) of the lavatory surfaces and 7.2 % (4/55) of the other surfaces (in total 5.1 %; 11/214). None of the hand swabs were positive for NoVs or eAdVs, but non-eAdV was detected in 18.8 % (6/32). The three fecal samples collected in 2014 were negative for NoVs and AdVs.

### Detection of the Process Control Viruses

The lower limit of an acceptable result for the process control virus detection by rRT-PCR was decided to be a C_t_ value < 40. In all expect four occasions, the positive control virus gave a positive result. We were not able to reanalyze the samples that remained negative for the process control viruses because no sample material remained after the initial nucleic acid extraction. The majority of the samples (94.1 %; data not shown) that were positive for NoVs and/or AdVs were, however, not the ones that were spiked with the process control viruses.

### Sequence Analysis

Of the total number of samples that were NoV-positive by rRT-PCR in 2013 and 2014 (*n* = 32; 25 surface swabs, two hand swabs, and five fecal samples), 17 surface and one hand swab sample collected in 2013, and one surface swab sample collected in 2014 did not show a right-sized product in any of the conventional RT-PCR-tests that targeted different regions of the genome, so these samples were not subjected to sequencing.

Partial NoV sequences from either the polymerase (ORF1) and/or capsid regions (ORF1/2 junction) were obtained from eight garrison A samples (Table 4). Regardless of the sample type (fecal or surface), all the GI.Pb-GI.6 sequences from six samples were 100 % identical. The two NoV GII-positive fecal samples represented different genotypes: sample F1 was a recombinant between the pandemic variants GII.P4-New Orleans-2009 and GII.4-Sydney-2012, while sample F4 represented genotype GII.7.

**Table 4:** Genotypes of the sequenced samples

Garrison	Sampling date (Year 2013)	Sample code	Sample type	Genotype			Genbank accession no.	
				ORF1^a^	ORF1/2^b^	ORF2^c^	ORF1	ORF1/2
A	Mar.5	F1	Fecal	GII.P4-New Orleans-2009	GII.4-Sydney-2012		KT943510	Identical to KT943512
	Mar.6	F2	Fecal	GI.Pb	GI.6	GI.6	Identical to KT943508	KT943509
	Mar.6	F3	Fecal	GI.Pb	GI.6	NA	Identical to KT943508	Identical to KT943509
	Mar.7	F4	Fecal	NA	GII.7	NA	NA	KT943513
	Mar.8	F5	Fecal	GI.Pb	GI.6	NA	Identical to KT943508	Identical to KT943509
	Mar.12	S1	Surface swab	GI.Pb	GI.6	GI.6	KT943508	Identical to KT943509
	Mar.12	S2	Surface swab	NA	NA	GI.6	NA	NA
	Apr.9	S3^d^	Surface swab	NA	GI.6	GI.6	NA	Identical to KT943509
B	Apr.10	S4	Surface swab	NA	GII.4-Sydney-2012	NA	NA	KT943511
	Apr.10	S5	Surface swab	NA	GII.4-Sydney-2012	NA	NA	KT943512
	Apr.17	S6	Surface swab	NA	GII.4-Sydney-2012	NA	NA	Identical to KT943511
	Apr.17	S7	Surface swab	NA	GII.4-Sydney-2012	NA	NA	identical to KT943511
	Apr.17	H1^d^	Hand swab	NA	GII.4-Sydney-2012	NA	NA	Identical to KT943512

^a^Genotype and variant, if available, according to the ORF1 sequence obtained with primers RegA and MJV12

^b^Genotype and variant, if available, according to the ORF1/2 junction sequence obtained either with primers JJVMF/G1SKR (samples F2, F3, F5, S1, S3) or QNIF2D/G2SKR (samples F1, F4, S4 – S7, H1)

^c^Genotype according to the ORF2 sequence obtained with primers GI.6RR/FF. These sequences were not submitted to the GenBank database

^d^The ORF1/2 junction sequences of the samples S3 and H1 were 198 bp and 127 bp, respectively

Partial NoV capsid sequences (ORF1/2 junction) were obtained from five garrison B samples (Table 4). The capsid sequences of the surface samples S4, S6, and S7 were 100.0 % identical with each other, and also with the short sequence obtained from the hand swab sample H1. This variant was identified as the NoV GII.4-Sydney-2012 by the RIVM norovirus genotyping tool (Kroneman et al. 2011). The capsid region of the other detected NoV GII.4-Sydney-2012 variant (S5) was 97.2 % identical with the surface samples S4, S6, and S7 but 100 % identical with the capsid region of the garrison A fecal sample F1. The exact variants of the samples F1 and S5 were not identified by the RIVM norovirus genotyping tool (Kroneman et al. 2011), but according to the NCBI BLAST, they were 100 % identical with the capsid region of a recombinant strain New Orleans 2009/Sydney 2012 (GenBank accession no. KF378731) that was detected in Italy in 2013 (Martella et al. 2013).

### Questionnaire Results

In 2013, all 28 conscripts in garrison A reported themselves as healthy (i.e., no AGE symptoms within 6 days at the time of the hand swabbing), but 28.6 % (8/28) of them had been in contact with another conscript who had AGE symptoms within the previous 6 days (Table 5). In contrast, 30.6 % (15/49) of the conscripts in garrison B in 2013 had suffered from AGE symptoms within 6 days before hand swabbing, and 63.3 % (31/49) of them had been in contact with another conscript who had suffered from AGE symptoms recently. Also, the conscripts in garrison B in 2013 had more contacts with non-military persons suffering from AGE symptoms than the conscripts in garrison A (10.7 vs. 40.8 %; *P* = 0.005, Mid-P exact test) in the same year. Recent AGE symptoms were rarer among the garrison B conscripts in 2014 when compared to their counterparts in 2013 (30.6 vs. 9.4 %; *P* = 0.025, Mid-P exact test). The conscripts’ contacts with other people (military or non-military) suffering from recent AGE symptoms did not differ significantly between years 2013 and 2014 in garrison B.

**Table 5:** Conscripts’ reports of their recent acute gastroenteritis (AGE) symptoms (diarrhea and either abdominal pain, vomiting, or both) and contacts with other conscripts or non-military persons suffering from AGE. Gar = garrison

Category	Year						*P* values^a^	
	2013				2014			
	Gar A	95 % CI	Gar B	95 % CI	Gar B	95 % CI	1	2
Conscripts who had AGE symptoms within 6 days before hand swabbing (no./total; %)	0/28 (0.0)	0.0–14.3	15/49 (30.6)	19.4–44.6	3/32 (9.4)	2.5–25.0	<0.001*	0.025*
Conscripts who had been in contact with another conscript who had AGE symptoms within 6 days before hand swabbing (no./total; %)	8/28 (28.6)	15.1–47.2	31/49 (63.3)	49.2–75.4	15/32 (46.9)	30.9–63.6	0.004*	0.156
Conscripts who had been in contact with non-military persons who had AGE symptoms within 6 days before hand swabbing (no./total; %)	3/28 (10.7)	2.9–28.0	20/49 (40.8)	28.2–54.8	9/32 (28.1)	15.4–45.5	0.005*	0.257

^a^ *P* values were calculated with mid-P exact test. *P* value 1 is calculated between the questionnaire results collected in garrisons A and B in 2013. *P* value 2 is calculated between the questionnaire results collected in garrison B in 2013 and 2014

* *P* values < 0.05 were considered statistically significant

Due to the low number of NoV- or eAdV-positive hand swabs, reliable statistical analysis between these findings, and the occurrence of AGE symptoms could not be performed. However, one of the conscripts that had NoV GII on his hands also had AGE symptoms within 6 days before the hand swabbing, whereas the other conscript who gave a NoV-positive hand swab, although not having AGE symptoms, had to be given intravenous fluids at the time of swabbing to treat dehydration. The conscripts that had eAdV on their hands did not report of recent AGE symptoms. Non-eAdV findings were not correlated with AGE symptoms, as expected.

## Discussion

This study revealed that NoV was present on the environmental surfaces of two Finnish garrisons for several weeks in spring 2013. During the first visit to each garrison, one quarter of the surface swabs were NoV-positive, which is in line with other studies that have been conducted during, or shortly after, an identified NoV outbreak (Cheesbrough et al. 2000; Wu et al. 2005; Jones et al. 2007; Wadl et al. 2010; Fankem et al. 2014). In contrast, NoV contamination on the surfaces was rare in January–February 2014. These results seem to reflect the overall NoV situation in Finland during our study periods, because in March–May 2013, the health authorities of Finland reported twice as many laboratory-confirmed NoV cases as they did in January–February 2014 (THL 2015). Although the detection of viral genome does not necessarily indicate the presence of infectious virus, NoV is known to be relatively stable on environmental surfaces (D´Souza et al. 2006), so transmission of viruses via fomites may have occurred. Also, Boxman et al. (2011) showed that even when there was no evidence of an ongoing NoV outbreak, surface contamination by NoV correlated with the food producing facility’s NoV outbreak history.

After our swab sampling period was finished in May 2013, we were informed that the personnel of garrison A had suspected a gastroenteritis outbreak, and collected 11 fecal samples from conscripts suffering from gastroenteritis in early March. Their suspicions were later supported by the detection of NoV in five of the fecal samples. Also, it was the same genotype (GI.6-GI.Pb) that was detected on the surfaces—including a surface in the sick bay—and in three of the fecal samples. Somewhat surprisingly, the questionnaires collected in this garrison did not indicate that gastroenteritis cases had occurred recently. However, these questionnaires and the hand swabs were collected several weeks later than most of the positive surface samples. Moreover, most of the conscripts who participated in the hand swab and questionnaire study were residing in living quarters which were not swab sampled.

In contrast, the questionnaires collected in garrison B clearly indicated that gastroenteritis cases had occurred during the study period: almost one-third (30.6 %) of the conscripts had AGE symptoms within 6 days before the hand swabbing, and the majority (63.3 %) of them had been in contact with other conscripts who were suffering from AGE. Unfortunately, we were not informed if the health care personnel of garrison B had suspected a gastroenteritis outbreak in spring 2013, and no fecal samples were obtained. NoV was, however, detected in two of the hand swabs collected in this garrison. According to Boxman et al. (2009) and Liu et al. (2013), infected persons often have detectable NoV on their hands, both in laboratory and outbreak settings. It has also been demonstrated that NoV remains detectable on finger pads only for a couple of hours (Liu et al. 2009), which implies that NoV contamination on the hands of these conscripts must have happened soon before the hand swabbing. The presence of the same NoV GII.4 variant both on the surfaces and in a hand swab further supported that NoV was at least one of the causative agents of these gastroenteritis cases.

Although NoV was detected on the surfaces of both garrisons for several weeks in 2013, NoV contamination on the same surface during two consecutive visits happened only twice. This implies that the surfaces had been cleaned and these surfaces were then recontaminated, either by new cases or by prolonged shedding of NoV by the recovered or asymptomatic cases. The spread of NoV via lavatory surfaces is a known risk. However, the frequent presence of NoV and AdV on several other environmental surfaces on the same premises suggest inadequate hygiene practices. Virus transmission via hands or fomites was therefore also possible in other facilities, recreational or otherwise. It has been reported that viral contamination can spread via contaminated cleaning equipment (Fankem et al. 2014), but it seems that the cleaning procedures in the two garrisons we studied were adequate for inactivating and removing NoVs from surfaces.

In our study, the presence of eAdV did not coincide with that of NoV. Non-eAdVs were, however, frequently present both on the surfaces and hands. The non-eAdVs on the surfaces were distributed between the lavatories and the other places similar to that found for NoVs; however, because some non-eAdVs are also excreted in feces (Russell et al. 2006; Lynch et al. 2011; Rusiñol et al. 2014; Verani et al. 2014), it is not possible to tell whether the source of non-eAdV was contamination from feces or from other bodily excretions. Other studies have also reported of detecting non-eAdVs frequently on lavatory surfaces and air (Russell et al. 2006; Verani et al. 2014). The prevalence of non-eAdV on the hands of conscripts was somewhat lower than that reported by Russell et al. (2006); in their study, 69 % of conscripts with febrile respiratory AdV illness had AdV 4 DNA on their hands. In our study, the conscripts were not questioned about recent symptoms of respiratory-or other illnesses, so based on our results we cannot exclude the possibility that an outbreak of non-eAdV was ongoing.

We conclude by stating that NoV cases occurred in both garrisons during the study period in 2013, and the detection of NoV on the surfaces during the same period was frequent. This was in contrast to the 2014 results, when both AGE cases and NoV findings on the surfaces were rare. We were not able to draw any conclusions on whether there was a correlation between the viral findings on hands and AGE symptoms because of the low number of NoV- or eAdV-positive hand swabs. Some swab samples remained negative for the process control viruses, which indicates that viruses are lost during sample processing. Therefore, it is possible that some of our swabs were false-negative for NoV and AdV. We find that routine surface swabbing, however, provides valuable information on the presence of both of these viruses, and we believe that in our study, the rapidly disseminated information of the virus-positive surfaces to the garrisons’ personnel had a role in preventing larger scale outbreaks caused by NoV.

## Electronic supplementary material

Below is the link to the electronic supplementary material.

Supplementary material 1 (PDF 216 kb)
