# Supplementary material for: Contamination by Norovirus and Adenovirus on Environmental Surfaces and in Hands of Conscripts in Two Finnish Garrisons
Source: Food Environ Virol. 2016 Sep 30;9(1):62–71. doi: 10.1007/s12560-016-9262-4 (PMC5309312; doi:10.1007/s12560-016-9262-4)
Supplement: Supplementary file 1 — Supplementary material 1 (PDF 216 kb) [file 12560_2016_9262_MOESM1_ESM.pdf]

Online Resource 1

Journal: Food and Environmental Virology

Article Title: Contamination by Norovirus and Adenovirus on Environmental Surfaces and in Hands of Conscripts in Two Finnish Garrisons

Authors: Satu Oristo, Maria Rönnqvist, Mika Aho, Ava Sovijärvi, Tuula Hannila-Handelberg, Ari Hörman, Simo Nikkari, Paula M. Kinnunen, and Leena Maunula.

Corresponding author: Satu Oristo, Department of Food Hygiene and Environmental Health, University of Helsinki, Finland. satu.oristo@helsinki.fi

Online Resource 1. The primers and probes used in this study

Assay	Target virus	Oligonucleotide	Sequence and label (5' → 3')	Nucleotide position	Reference
rRT-PCR	NoV GI[a]	QNIF4	CGCTGGATGCGNTTCCAT	5291 – 5308 [b]	da Silva et al. 2007
		QNIF3	GTCCTTAGACGCCATCATCATTT	5356 – 5378 [b]	Summa et al. 2012
		JJV1P	FAM-TGTGGACAGGAGATCGCAATCTC-BHQ1	5319 – 5342 [b]	Jothikumar et al. 2005b
	NoV GII	QNIF2D	ATGTTCAGRTGGATGAGRTTCTCWGA	5012 – 5037 [c]	Loisy et al. 2005
		COG2R	TCGACGCCATCTTCATTCACA	5080 – 5100 [c]	Kageyama et al. 2003
		QNIFS	FAM-AGCACGTGGGAGGGCGATCG-BHQ1	5042 – 5061 [c]	Loisy et al. 2005
	MeV	Mengo 110	GCGGGTCCTGCCGAAAGT	110 – 127 [d]	Pintó et al. 2009
		Mengo209	GAAGTAACATATAGACAGACGCACAC	184 – 209 [d]	Pintó et al. 2009
		Mengo147	ATCACATTACTGGCCGAAGC	147 – 166 [d]	Pintó et al. 2009
	MuNoV	MNV for	TGCAAGCTCTACAACGAAGG	6520 – 6539 [e]	Hewitt et al. 2009
		MNV rev	CACAGAGGCCAATTGGTAAA	6626 – 6645 [e]	Hewitt et al. 2009
		MNV pro	TAMRA-CCTTCCCGACCGATGGCATC-BHQ1	6578 – 6597 [e]	Hewitt et al. 2009
rPCR	AdV	JTVXF	GGACGCCTCGGAGTACCTGAG	54 – 74 [f]	Jothikumar et al. 2005a
		JTVXR	ACIGTGGGGTTTCTGAACTTGTT	127 – 149 [f]	Jothikumar et al. 2005a
		JTVXP	FAM-CTGGTGCAGTTCGCCCGTGCCA-BHQ1	82 – 103 [f]	Jothikumar et al. 2005a
	AdV 40/41	AdV Fs	CTCGACATGACTTTTGAGGT	2614 – 2633 [f]	van Maarseveen et al. 2010
		AdV Fas	GTAGACGGCCTCGATGAC	2716 – 2733 [f]	van Maarseveen et al. 2010
		AdV Pro	AGCCCACACTTCT	2648 – 2660 [f]	van Maarseveen et al. 2010
RT-PCR	NoV GI	JJVMF	CCATGTTCCGTTGGATGC	5283 – 5300 [b]	Hill et al. 2010
		G1SKR	CCAACCCARCCATTRTACA	5653 – 5671 [b]	Kojima et al. 2002
	NoV GI.6	GI.6 FF	AGCTGGTACCGGAGGCTAAT	5413 – 5432 [g]	This study
		GI.6 RR	AACAAAATGTCACCGGGGGT	5577 – 5596 [g]	This study
	NoV GII	QNIF2D	ATGTTCAGRTGGATGAGRTTCTCWGA	5012 – 5037 [c]	Loisy et al. 2005
		G2SKR	CCRCCNGCATRHCCRTTRTACAT	5367 – 5389 [c]	Kojima et al. 2002
	NoV GI, NoV GII	MJV12	TAYCAYTATGATGCHGAYTA	4553 – 4572 [b] 4280 – 4299 [c]	Vinjé et al. 2004
		RegA	CTCRTCATCICCATARAAIGA	4859 – 4879 [b] 4586 – 4606 [c]	Vinjé et al. 2004

[a] NoV GI: Norovirus genogroup I, NoV GII: Norovirus genogroup II, MeV: Mengovirus, MuNoV: Murine norovirus, AdV: Adenovirus, AdV 40/41: Adenovirus types 40 and 41

[b] Positions based on complete NoV GI genome (GenBank accession no. M87661)

[c] Positions based on complete NoV GII genome (GenBank accession no. X86557)

[d] Positions based on complete MeV genome (GenBank accession no. L22089)

[e] Positions based on complete MuNoV genome (GenBank accession no. AY228235)

[f] Positions based on AdV hexon gene (GenBank accession no. X51782)

[g] Positions based on complete NoV GI.6 genome (GenBank accession no. JQ388274)
